# The regenerative potential of Pax3/Pax7 on skeletal muscle injury

**DOI:** 10.1186/s43141-022-00429-x

**Published:** 2022-10-17

**Authors:** Muhamad Azhar, Bantari Wisynu Kusuma Wardhani, Editha Renesteen

**Affiliations:** Faculty of Military Pharmacy, The Republic of Indonesia Defense University, Bogor, 16810 West Java Indonesia

## Abstract

**Background:**

Skeletal muscle mishaps are the most well-known incidents in society, especially among athletes and the military population. From the various urgency, this accident needs to be cured more quickly. However, the current treatment still has some shortcomings and is less effective. In this case, Paired box 3 and Paired box 7 (Pax3/Pax7) proteins that induce stem cells could potentially be an alternative treatment for skeletal muscle injuries. This paper aimed to analyse the potential treatment of Pax3/Pax7 proteins inducing the stem cell for skeletal muscle injuries.

**The main body of the abstract:**

We did a narrative review by gathering several scientific journals from several leading platforms like PubMed and Scopus. As common accidents, skeletal muscle disease could be due to workplace and non-workplace causes. The highest risk occurs in the athlete and military environment. The treatment of current skeletal muscle injuries is protection, rest, ice, compression, and elevation (PRICE), non-steroidal anti-inflammatory drugs (NSAIDs), and mechanical stimulation. However, it is considered less effective, especially in NSAIDs, inhibiting myogenic cell proliferation. The current finding indicates that the stem cells have markers known as Pax3/Pax7. The role of both markers in muscle injury, Pax3/Pax7, as transcription factors will induce cell division by H3K4 methylation mechanisms and chromatin modifications that stimulate gene activation.

**Conclusion:**

Regulation by Pax3/Pax7 factors that affect stem cells and stem cell proliferation is one of the alternative treatments. This regulation can accelerate the healing of injury victims, especially injuries to the skeletal muscles. Finally, after being compared, Pax3/Pax7 induces stem cells to have the potential to be one of the skeletal muscle injury treatments.

**Keywords:**

Pax3 and Pax7, Pax3/Pax7, Skeletal muscle, Athlete, Stem cells, Cell proliferation, Injuries.

## Background

Skeletal muscle comprises about 40% of our weight and multinucleated contractile muscle strands [1, 2]. Skeletal muscle functions for the body to move and undertake activities, as well as maintain vital organs in our body [2, 3]. Therefore, it is easier to get injuries, especially for groups who conduct much physical activity, such as athletes and military personnel [4–7]. In a retrospective study, 65 of 79 gymnastic athletes experienced skeletal muscle injuries [8]. In addition, in the military environment, American soldiers in Afghanistan, as many as 35% of 15,000 soldiers experienced skeletal muscle injuries [9].

As the population is most commonly affected, skeletal muscle injuries can impact the productivity and performance of athletes and military personnel in conducting their duty. Hence, the recovery stage needs to be completed immediately and effectively [5]. Current therapies for skeletal muscle injuries are protection, rest, ice, compression, and elevation (PRICE or RICE) principles, pharmacological intervention such as non-steroidal anti-inflammatory drugs (NSAIDs), surgical treatment, and mechanical stimulation [10–12]. PRICE can stop intramuscular bleeding and muscle injuries [7, 13], while pharmacological intervention with NSAIDs can decrease the inflammatory cell reaction to recover the injury [14]. Likewise, surgery can be implemented in specific conditions and may also be considered if the patient has persistent pain for more than 4 to 6 months [13]. Finally, mechanical incitement can expand the recovery of the seriously harmed skeletal muscle, lessening the requirement for exogenous or cell development factors [15].

Despite these existing treatments, current therapies still have several drawbacks. For example, PRICE or RICE is still not clinically proven in randomised control trials (RCTs) studies; likewise, a long treatment period can increase bleeding [7]. When using higher doses of NSAIDs, many side effects may arise, and delayed muscle regeneration can be developed [7, 14]. Another therapy option with surgical treatment should be carried out under certain circumstances, and only the cost is quite expensive. A previous study stated that after surgery, patients could feel pain and irritation [7, 11]. When treating skeletal muscle injuries with mechanical stimulation, this treatment can enlarge the scar and continue the rupture [7]. Therefore, stem cell therapy can be another option to tackle these constraints.

Stem cell is one of the areas of interest in health and contributes to treating diseases. As an alternative treatment in skeletal muscle, stem cells have functional marker proteins in cell proliferation, particularly Pax3 and Pax7 (Pax3/Pax7). In one study, this protein strongly influences stem cell division [16, 17]. Hence, it is necessary to comprehend the function of the proteins themselves to medicate the disease.

Taking together those reasons, the purpose of this review is in its application, and it should be noted that these proteins can be used as an alternative treatment for skeletal muscle injuries. We will explain the mechanism and value of this protein innovation on gene regeneration and compare it with previous treatments.

## Main text

### The cause of skeletal muscle injuries

**Fig. 1:**
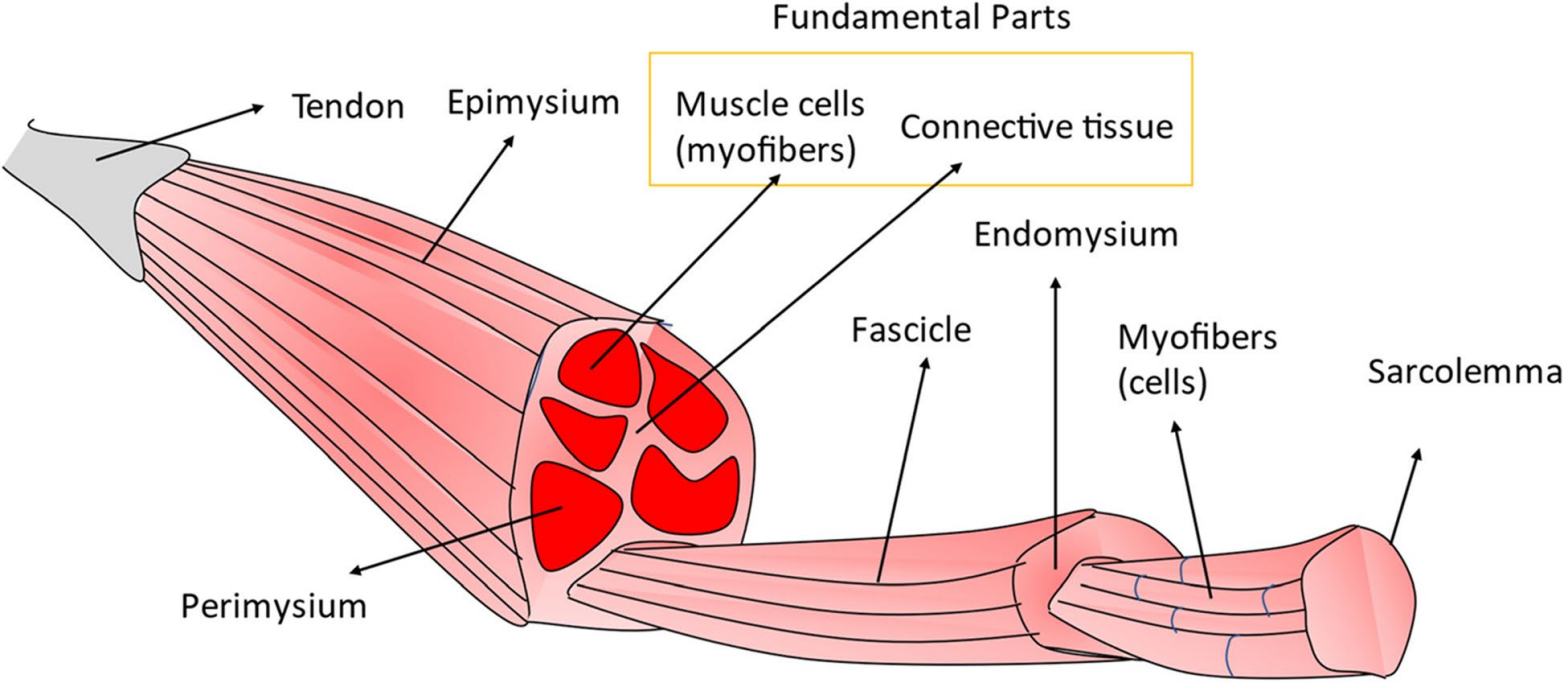
Skeletal muscle anatomy comprises two fundamental parts: myofibres and connective tissue

Skeletal muscle fibres originate from bone and connective tissue inserted through tendons (Fig. 1) [13]. Skeletal muscle comprises two fundamental parts, myofibres and connective tissue [11, 14]. Skeletal muscle functions for the body to move and perform activities, as well as maintain vital organs in our body [2, 3]. Hence, the skeletal muscles are essential to body parts [18].

Skeletal muscle injuries are common traumas in many populations, especially in sports and the military [4, 5, 13, 14]. This type of injury usually involves various severity and events, both direct and indirect trauma [1]. In the sports population, the incidence varies from 10 to 50% [14], while in the military, another study mentioned that non-battle injury could reach 77% of the 3300 American soldiers [19]. Of the two populations, the body parts that suffered the most injuries were the low back (17%), knee (12%), shoulder (10%), and most of the time when working (65%) [5].

Several factors that cause skeletal muscle injuries are sprains, bruises, and lacerations. Furthermore, more than 90% of incidents associated with sports are bruises and sprains [13]. The causes of skeletal muscle injuries are divided into two factors, particularly workplace and non-workplace factors. Workplace factors such as climbing, lifting, and overwork are associated with biomechanical risk factors, individual predispositions, and psychosocial conditions [20], while non-workplace factors such as physical stress and even comorbidities cause skeletal muscle injuries [6, 20]. Among athletes and the military population, these causes of skeletal muscle injuries are divided into two major factors, in particular intrinsic factors, such as age, sex, anatomy, and physical activity, as well as extrinsic factors, such as the amount of training, the type of training, and the acceleration of training [5, 6]. Meanwhile, there are more causes of skeletal muscle injuries from retrospective studies summarised in Table 1.

**Table 1:** The cause of skeletal muscle injuries

Cause	Summary	Mechanism	Reference
Physics	It is divided into workplace factors and non-workplace factors	Both factors cause repetitive and hard muscle work. Muscles will be injured, causing acute and systemic inflammation. Fibrosis will appear and cause the muscles to lose function	[5, 6, 13, 20]
Virus	SARS-CoV-2 virus, Zika virus (ZIKV), Chikungunya (CHIK), and Influenza A have been reported to cause injuries to skeletal muscles with tissue damage mechanism that causes myotoxicity	The SARS-CoV-2 virus can affect skeletal muscle by downregulating angiotensin-converting enzyme 2 (ACE-2) receptors, increasing angiotensin (Ang) II levels, and reducing Ang 1–7	[21–25]
Bacteria	*Clostridium perfringens* bacteria can cause a deficiency in skeletal muscle regeneration after injury	*Clostridium perfringens* bacteria can exacerbate skeletal muscle injuries, leading to myonecrosis due to the inflammatory factor IFN-γ	[26]
Chemical	The oxime group can cause acute skeletal muscle injury	Chemicals such as oxime can cause acute skeletal muscle injury by necrotising and inflammatory mechanisms	[27]

Some factors are known to cause skeletal muscle injuries, for example, the presence of viral pathogens. Recently, the SARS-CoV-2 virus can cause serious injury to skeletal muscle by downregulating angiotensin-converting enzyme 2 (ACE-2) receptors, increasing angiotensin (Ang) II levels, and reducing Ang 1–7. The SARS-CoV-2 virus can cause muscle damage when these levels drop. Another viral mechanism leading to skeletal muscle injuries is inducing inflammation in skeletal muscle. A previous study by Lentscher et al. created an engineered Chikungunya virus (CHIK) strain that exhibits in-cell replication via the miR-206 target sequence. They found that significantly reduced inflammation and necrosis in muscles arose when CHIKV replication in skeletal muscle cells was restricted. Another study by Gavino-Leopoldino et al. created a mouse model of Zika virus (ZIKV), and its infection induces necrotic lesions, inflammation, and fibre atrophy [21–25].

Another cause of muscle injuries, bacterial infections, such as *Clostridium perfringens* bacteria, can exacerbate skeletal muscle injuries, leading to myonecrosis due to the inflammatory factor IFN-γ. Zúñiga-Pereira et al., in their study, characterised the process of muscle regeneration after myonecrosis in experiments on murine infected with *C. perfringens*. This bacterial pathogenicity causes reduced muscle regenerative activity [26]. Finally, chemicals such as oxime can cause acute skeletal muscle injury by necrotising and performing inflammatory mechanisms. In one study, oxime was tested on mice leading to changes in the morphology. Muscle degeneration occurred when given treatment with a dose of 0.5 LD50, and acute toxic muscle injury developed in 7 days, resulting in necrosis [27].

### Current treatment for skeletal muscle injuries and their limitations

Skeletal muscle injury is the most common injury which goes through three phases: the acute inflammatory and degenerative phase, the repair phase, and the remodelling phase. Current conservative treatments include protection, rest, ice, compression, and elevation (PRICE), nonsteroidal anti-inflammatory drugs (NSAIDs), and physical therapy [7]. It turns out that NSAIDs are used to suppress the inflammatory phase in injury. Other therapies and strategies are growth factors, stem cells, scaffolds, anti-fibrotic therapy, and mechanical stimulation [10]. In several journals, it is stated that, in athletes and military populations, antioxidants are used to prevent necrosis and degeneration, growth factors to promote muscle healing, and NSAIDs to reduce inflammation [4, 28].

#### RICE or PRICE

RICE or PRICE or protection, rest, ice, compression, and elevation is a mechanical treatment to minimise the injury at the injury site and prevent the extension of trauma to adjacent muscle areas [11, 13]. This method is a traditional method and is not widely known, but it can still be used and is not overly threatening for injured muscles. This method begins with resting the wound for 7 days and physiotherapy, respectively [7]. Nevertheless, PRICE is still not clinically proven in RCT studies, while a long treatment period can increase bleeding [7].

#### NSAIDs

NSAIDs can interfere with the activity of macrophages and breaking point phagocytic, in any event, hindering the creation of development inciting factors liable for recovery after muscle injury [7]. This drug has become an option for reducing pain after an injury. However, the use of NSAIDs stays dubious for their utilisation in the treatment of muscle injury [4]. NSAIDs can lead to incomplete recovery and, when continuously used, can affect muscle regeneration and contractile function [11, 29]. Therefore, this pharmacological treatment can potentially inhibit muscle regeneration in long-term consumption [7].

#### Mechanical stimulation

This treatment is the simplest method for stimulating mechanics. Mechanical strength is a biological regulator as crucial as chemicals and genes and has excellent potential for the development of mechano-therapy to treat muscle damage [15]. In an in vitro study, the use of this method works in muscle growth, differentiation, and muscle function, as well as in controlled stimulation to increase myogenesis [30]. Furthermore, mechanical stimulation aids in enhancing cellular alignment and inducing the formation and growth of radial muscular fibres, mimicking the fibre orientation in a healthy setting [31–34].

### Pax3/Pax7 as an alternative therapy for skeletal muscle injury

A surrogate answer to recuperating skeletal muscle wounds is cell treatment since skeletal muscle is an optimal objective for targeting cell treatment [29]. The purpose of cell therapy is to work on the structure and capacity of muscle tissue [11]. The cells used are stem cells due to their strong dividing ability [35, 36].

Stem cells are characterised by the capability to expand and can be differentiated into various cell and tissue sources [16]. Stem cells are differentiated into a few characterisations dependent on differentiation potential, one of which is unipotent cells, which are cells that can only be differentiated into one specific cell type, such as muscle cells [16]. Based on the source, adult stem cells are unipotent. Thus, the skeletal muscle stem cells in adult bodies have become satellite cells [37].

Skeletal muscle regeneration requires important cells to regenerate, for instance, satellite cells and skeletal stem cells [38]. The candidate cells are myogenic cells and non-myogenic cells [11]. The myogenic stem cell method occurs when muscle-derivate stem cells (MDSCs) are transplanted, forming myofibres grafted onto the damaged area [11, 35]. Different types of cells do not consistently have similar differentiation abilities. The use of other stem cells can also be used with non-myogenic cell methods, such as using mesenchymal stem cells (MSCs) or adipose-derived stem cells (ADSCs) [39, 40].

Satellite cell-dependent myogenesis is a complex process characterised by the muscle-specific transcription factor Pax3/Pax7 from MyoD members [36]. Pax genes, especially Pax7, are significant in muscle development [41, 42]. When skeletal muscle is injured, the intervention of Pax3/Pax7 genes will continue to increase and begin to regulate cell division to cover the injury [41, 43]. The combination of several transcription factors with Pax3/Pax7 can induce skeletal muscle stem cells by activating transcription the cell proliferation [44, 45].

### The mechanism of action of Pax3/Pax7 on skeletal muscle stem cell

#### Cellular mechanism of Pax3/Pax7 in cell proliferation: regulation of gene expression

Generally, skeletal muscle regeneration is divided into three stages, in particular, an inflammatory phase caused by muscle damage; satellite cell activation due to proliferation; and the maturation phase of differentiation, the process of forming new myofibres. In the first stage, muscle cells will degenerate due to damage. Inflammation goes hand in hand with the immune response due to muscle tissue damage. Inflammation begins with the innate immune system response, induced by damage-associated molecular patterns (DAMPs) released by necrotic cells. DAMPs activate immune cells, such as mast cells, monocytes, and macrophage cells. These cells release pro-inflammatory cytokines and chemokines, causing activation of T-lymphocytes, either T helper (Th) or cytotoxic T cells, and circulating these lymphocytes to the injury site. Activated Th cells release inflammatory factors, causing vasodilation such as interferon γ (IFN-γ). Cytotoxic T cells can induce tissue damage, aggravating muscle injury [37, 46]. In the second stage, satellite cells are described by Pax7 articulation but do not express myogenic differentiation (MyoD). However, in a state of injury, this stage is done to prioritise entry into the cell cycle, begin to express MyoD and relocate to the injury site, and combine and become ancestor myogenic cells [38]. The third stage is maturation into myofibres which become the contractile units of skeletal muscle.

**Fig. 2:**
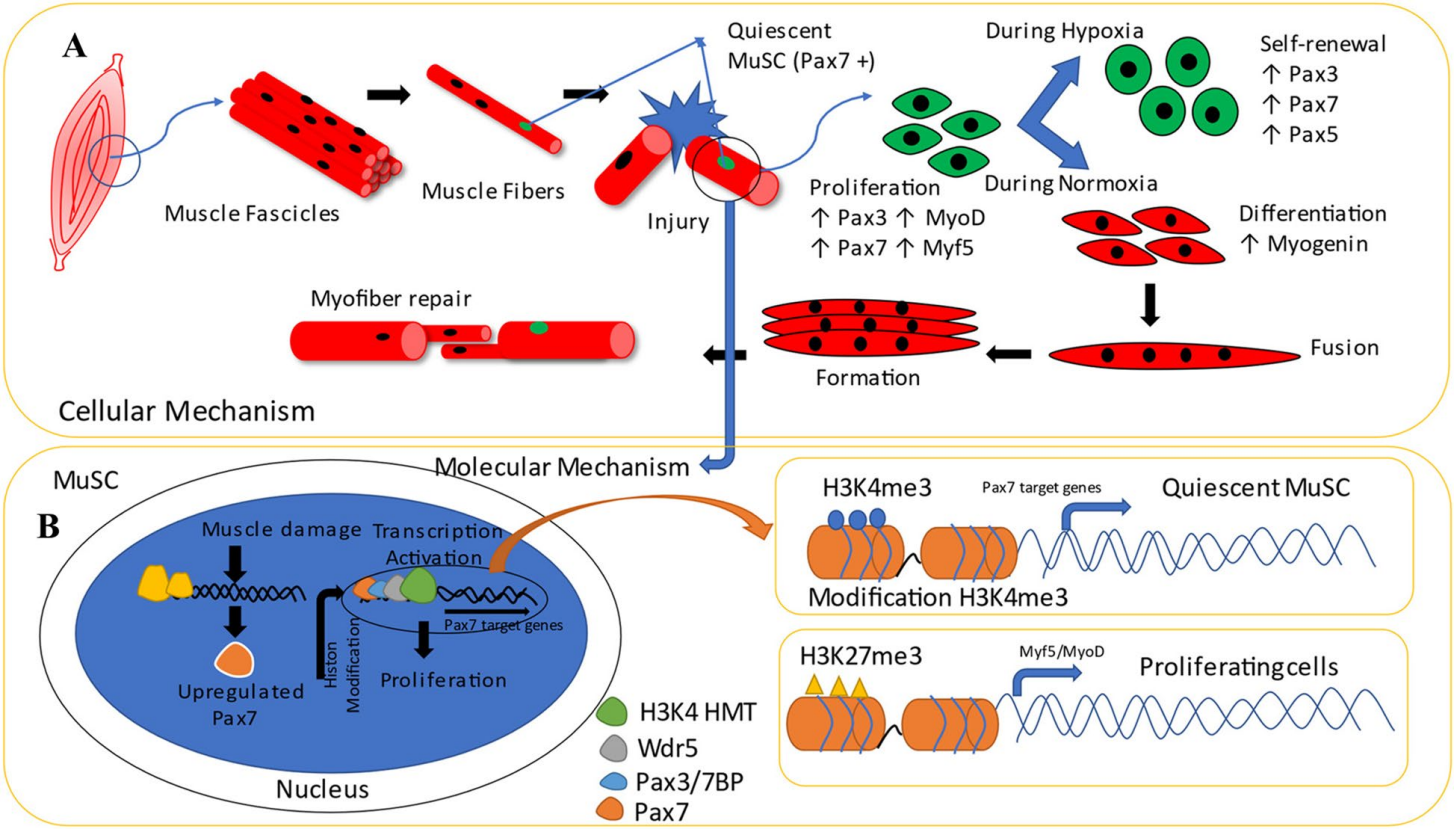
The mechanism of action of Pax3/Pax7 for skeletal muscle stem cells is divided into two mechanisms; cellular and molecular mechanisms. **A** Cellularly, the mechanism consists of six stages: (1) muscle damage, (2) proliferation, (3) differentiation, (4) fusion, (5) formation, and (6) myofibre repair. **B** Molecularly, the mechanism is divided into several stages: (1) histone modification and (2) proliferation activation against the recruitment of a histone methyltransferase complex (HMT)

In general, satellite cells are the primary support in the recovery of skeletal muscle cells [38]. Pax3/Pax7 is conveyed by the satellite cells and directed during myogenic separation. The existence of Pax3/Pax7 helps myogenic factor 5 (Myf5) and has a positive–negative function in cell proliferation [47]. Pax3/Pax7, together with Myf5, controls the input when starting the cell programme. If these three genes are not present, cell differentiation will not exist. Pax3/Pax7 assume an equal part in MyoD initiation, resulting in skeletal muscle separation [41]. Finally, Pax3/Pax7 can control the occurrence of division and inhibit cell differentiation (Fig. 2) [41, 48].

#### Molecular mechanism of Pax3/Pax7 on skeletal muscle stem cell

##### Transcription factors in myogenic genes

Pax3/Pax7 are the transcription factors of the specified muscle cells characterised. During the undeveloped turn of events, the transcription factors Pax3/Pax7 are viewed as significant controllers in the detail of muscle and tissue-framing cells [36]. However, in grown-up satellite cells, Pax7 is deeply overexpressed, and Pax3 is poorly expressed in the muscle area [36].

As explained above, the muscle regeneration process in stage two activates satellite cells. The transcription factor is viewed as a marker in fixed satellite cells, and Myf5 is the objective of transcription factor Pax7 once initiated. Activated satellite cells assert Pax7 and Myf5 and enter a proliferative state because Pax7 induces histone modification that stimulates transcription activation to regulate myogenic development [17, 36].

##### Proliferation activation against the recruitment of a histone methyltransferase complex (HMT)

In general, Pax7 specifically binds to the methylation regulatory element H3K4 in the target gene leading to the creation of the HMT core complex, for example, the strong trimethylation of H3K4 from the regulatory element, thereby activating subsequent gene expression. Another circumstance shows communication between Wdr5-Ash2L and Pax7 with the association of Pax7 with the HMT complex. Pax7 induces satellite cells by recruiting the HMT complex onto Myf5 by generating transcription activation [17]. The Pax3/Pax7 binding protein (Pax3/Pax7 BP) links this binding, so HMT-bound Pax7 is dependent on Pax3/Pax7 BP [49]. Thus, Pax7 can induce chromatin modification that stimulates the activation of target genes so that cells enter into the myogenic development programme [17].

### The role of Pax3/Pax7 in hypoxia

**Fig. 3:**
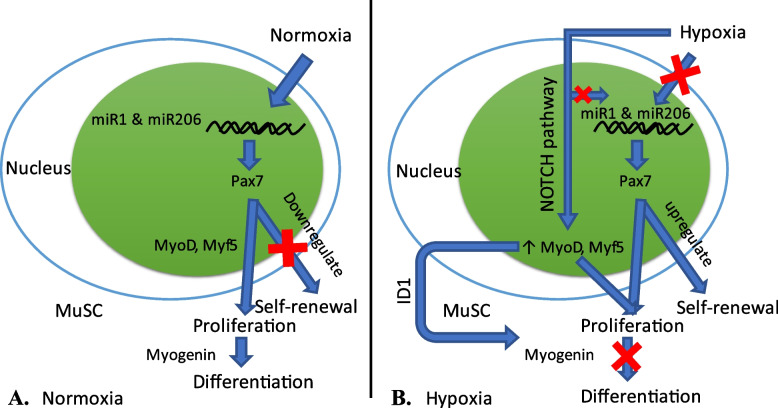
The role of Pax 7 on the proliferation of skeletal muscle cells in the condition of **A** normoxia and **B** hypoxia

During normoxia, Pax7 is downregulated by microRNA1 and 206 (miR1 and miR206), leading to decreased self-renewal of the satellite cells (Fig. 3A). While in hypoxia, the expression of miR1 & miR206 will be reduced, leading to the upregulation of asymmetric self-renewal division of satellite cells by Pax 7. This process mainly occurs due to the activation of the NOTCH signalling pathway (Fig. 3B). Hypoxic conditions can stimulate cell division in human primary myoblasts by increasing the expression of myogenic transcription factors Myf5 and MyoD1 [50]. Not only escalate the level of Myf-5 and MyoD1, but hypoxia also increases the level of myogenin which play a pivotal role in cell differentiation. The level rise occurs because hypoxia can induce H2K27 methylation and activate hypoxia-inducible factor 1-alpha (HIF1A) so that myogenesis factor expression is reduced [50, 51].

The role of Pax7 appears when its levels increase in hypoxia conditions. Pax7 will initiate a satellite cell asymmetric self-renewal programme and a cell proliferation [50, 52]. This mechanism aligns with the previous study, which indicated that 48 h under hypoxia, proliferation, and motility of skeletal muscle stem cell was enhanced. The number of undifferentiated cells positive for Pax 7 was increased at 72 h after hypoxia [52].

### In vitro and in vivo studies of Pax3/Pax7

Previous in vitro and in vivo studies have been directed to acquire the mechanical properties of Pax3/Pax7. For example, one in vitro study using primary myoblast cells said that the prevailing job of Pax7 is because of high homeobox partiality in grown-up myogenesis. Moreover, one of the in vivo studies using mice was injected with mutant embryonic stem cells (ESCs) that lost the Pax7 gene and said that the initiation of myogenic differentiation is facilitated while Pax7 is absent. Those studies are described and summarised in Table 2.

**Table 2:** Studies of Pax3/Pax7 using in vitro and in vivo models

	**Results**	**Reference**
**In vitro**		
**Cell line**		
Cell from muscle tissue of mutated mice (Pax3^nLacZ/+^ and Pax7^LacZ/+^ mice)	Progressive loss of Pax7 leads to satellite cell death during the cell cycle	[41]
Primary myoblast cells	In mature myoblasts, Pax3 binds to several subsets of Pax7 targets and thus has overlapping functions in the transcriptional network	[48]
iPax7 cells {pluripotent stem cells (PSC)}	Pax7 binding induces chromatin remodelling characterised by histone markers related to enhancers and acceptance of chromatin availability in PSCs	[53]
**In vivo**		
**Animal**		
Young mice were injected with siRNA	Studies have shown that H3K4 is deactivated by the knockdown of Pax3/Pax7 binding protein and restrains the multiplication of muscle precursor cells (MPCs). Therefore, the Pax3/Pax7 binding protein becomes the adapter that connects the Pax3/Pax7 with the H3K4 HMT	[49]
Mice were injected by ESCs (WT/KO of Pax7)	Initiation of myogenic differentiation is facilitated while Pax7 is absent	[54]
Mice were injected with TCDD	Pax3/Pax7 is needed to guard against TCDD (pollution)	[55]
Pax7 gene deprived mice (Pax7^−/−^ mice)	Pax3^+^ in muscle interstitials expresses MyoD during regeneration, and Pax7 functions in developing utilitarian myogenic progenitors from sublaminar satellite cells	[56]
Pax3^GFP/null^ mice crossing with Pax3^GFP/+^ mice	miR-27b directs Pax3 protein levels, and this guideline guarantees a quick and vigorous section into the myogenic separation programme	[57]
Mouse mutant	Pax + elicits muscle progenitors. There is no skeletal muscle; the mice experienced apoptosis after down-regulation of Pax7	[58]

*WT* Wildtype, *KO* Knocked-out, *TCDD* 2,3,7,8-tetrachlorodibenzo-p-dioxin

Despite in vitro and in vivo studies previously conducted, clinical studies that mention Pax3/Pax7 testing in skeletal muscle injuries are not yet available on related websites such as https://www.cochranelibrary.com/central/about-central and https://clinicaltrials.gov/.

## Conclusion

Stem cells characterised by Pax3/Pax7 proteins have great potential to become one the injury healing targets; drug substituents can be developed to target these marker proteins. This potential treatment could be seen from the ability of Pax3/Pax7 as transcription factors in stem cells to produce cell division proteins. The comparison between one study to another study indicates the mutant mice modified by the lacking Pax7 (Pax7−/−) were shown to decrease the growth and differentiation of skeletal muscle cells, leading to a lack of satellite cell function. These findings have a great potential to be developed as a new treatment; however, there are still limitations. The constraints of this study could be an opportunity for the state-of-the-art medication to treat skeletal muscle injuries. In addition, gene-edited test animals are needed to test their potency.

The comparison between current treatment (RICE or PRICE principles, NSAIDs, surgery, and mechanical stimulation) with alternative solutions with stem cells induced by Pax3/Pax7 resulted that the potential for stem cells induced by Pax3/Pax7 is very likely to be one of the treatments of skeletal muscle injuries.

Furthermore, it still needs to be studied further through in vitro and in vivo studies regarding the activity of Pax3/Pax7 that induce stem cells and the potential of these proteins for the treatment of skeletal muscle injuries. Assessment is also needed to find the best formulation for this treatment preparation. After studies in vitro and in vivo, it is hoped that this treatment will be developed into a clinical study and solve the current obstacle to treating athletes and military personnel who have a high incidence of skeletal muscle injury.

## Data Availability

There is no availability of data and materials.

## Abbreviations

ADSCs: Adipose-derived stem cells
ACE-2: Angiotensin-converting enzyme 2
Ang: Angiotensin
CHIK: Chikungunya virus
DAMPs: Damage-associated molecular patterns
ESCs: Embryonic stem cells
HIF1A: Hypoxia-inducible factor 1-alpha
HMT: Histone methyltransferase complex
IFN-γ: Interferon γ
LD50: Lethal dose of 50
MDSCs: Muscle-derivated suppressor cells
miR-1: MicroRNA 1
mir-206: MicroRNA 206
MSCs: Mesenchymal stem cells
MuCSs: Muscle stem cells
Myf5: Myogenic factor 5
MyoD: Myogenic differentiation
NSAIDs: Nonsteroidal anti-inflammatory drugs
Pax3: Paired box 3
Pax7: Paired box 7
Pax3/Pax7: Paired box 3 and Paired box 7
Pax3/Pax7BP: Pax3/Pax7 binding protein
PRICE: Protection, rest, ice, compression, and elevation
TCDD: 2,3,7,8-tetrachlorodibenzo-p-dioxin
Wdr5: WD repeat domain 5
ZIKV: Zika virus
